# Antiviral treatment for viral pneumonia: current drugs and natural compounds

**DOI:** 10.1186/s12985-025-02666-1

**Published:** 2025-03-06

**Authors:** Hao Zhang, Chunxia Ge, David Fisher, Nguyen Thi Thu Hien, Erkin Musabaev, Khrystyna Pronyuk, Yin Xia, Zhide Zhu, Yan Wang, Yiping Dang, Lei Zhao

**Affiliations:** 1 Institute of Medical Artificial Intelligence, Binzhou Medical University, Yantai, 264003 China; 2 Department of Medical Biosciences, Faculty of Natural Sciences, University of The Western Cape, Cape Town, South Africa; 3 Hai Phong University of Medicine and Pharmacy, Hai Phong, 04212 Viet Nam; 4 The Research Institute of Virology, Ministry of Health, 100122 Tashkent, Uzbekistan; 5 Infectious Diseases Department, O.Bogomolets National Medical University, Kyiv, 02132 Ukraine; 6 Department of Vascular Surgery, the Affiliated People’s Hospital of Fujian University of Traditional Chinese Medicine, Fuzhou, 350004 China; 7 The First Clinical Medical College, Guangxi University of Chinese Medicine, No. 89, Dongge Road, Nanning, 530023 Guangxi China; 8 Department of Vascular Surgery, Union Hospital, Tongji Medical College, Huazhong University of Science and Technology, Wuhan, 430022 China; 9 Department of Infectious Diseases, Union Hospital, Tongji Medical College, Huazhong University of Science and Technology, Wuhan, 430022 China

**Keywords:** Viral pneumonia, Antiviral drugs, Natural compounds, Review

## Abstract

In recent years, viral pneumonia has become a significant challenge to global public health, particularly during the COVID-19 pandemic. Viral pneumonia can be caused by various viruses, including influenza virus, RSV, and adenovirus. These viruses trigger inflammatory responses by invading the respiratory epithelial cells, leading to lung damage. Existing antiviral drugs such as ribavirin, adobiravir, and oseltamivir exert their therapeutic effects by inhibiting different stages of the viral life cycle but face issues such as increasing drug resistance. Natural components like astragalus saponins, Houttuynia cordata flavonoids, and tea theaflavin-gallates have demonstrated supportive roles in antiviral treatments, capable of not only enhancing immune responses but also potentially inhibiting viral replication through multiple pathways, thereby alleviating lung damage. Although natural components cannot entirely replace traditional antiviral drugs, their role in comprehensive treatment regimens is becoming increasingly important. This review summarizes the current applications and limitations of antiviral drugs and explores the research progress and potential mechanisms of natural components in the treatment of viral pneumonia.

## Introduction

In recent years, viral pneumonia has become a severe challenge in the field of global public health, especially during the COVID-19 pandemic, where outbreaks have garnered widespread attention [1]. The disease can be caused by a variety of viruses, including Influenza A and B viruses, Respiratory Syncytial Virus (RSV), Adenovirus, EB virus, and Cytomegalovirus [2]. Viruses invade the respiratory epithelium, triggering bronchiolitis, and subsequently spread to the alveoli and interstitial spaces of the lungs, leading to severe pulmonary inflammatory responses and impairment of respiratory function [3].

The mechanism of viral infection is complex, typically involving viral attachment, entry into host cells, uncoating, replication of viral nucleic acids, synthesis of viral proteins, assembly of viral particles, and their release [4, 5]. Based on the type of nucleic acid, viruses can be classified as RNA viruses or DNA viruses [6]. Targeting these steps, existing antiviral drugs exert their therapeutic effects by inhibiting key stages of the viral life cycle. However, with the emergence of resistant viral strains and the limitations of antiviral therapy efficacy, finding new treatment methods has become critically important.

The role of antiviral drugs in the treatment of viral pneumonia cannot be overlooked, especially when the condition progresses rapidly; antiviral therapy is an essential part of rescue treatment [7]. However, alongside these conventional therapies, natural components, particularly plant extracts, have gradually gained recognition for their potential as adjunctive treatments in recent years. These natural components not only demonstrate the ability to enhance the host’s immune response and modulate inflammatory reactions but may also inhibit viral replication through multiple pathways, reducing lung damage and improving patient recovery outcomes [8].

While natural components cannot replace existing antiviral drugs, their supportive role within comprehensive treatment protocols should not be underestimated. For instance, certain plant extracts have shown anti-inflammatory, antioxidant, and immunomodulatory activities [9–11], which are critical for adjunctive treatments of viral pneumonia. Therefore, exploring the mechanisms of action of these components and their potential synergistic use with existing antiviral drugs could provide new insights for future treatment strategies.

In recent years, significant progress has been made in the methods and approaches for discovering drugs for viral pneumonia, driven by advancements in computational biology, systems biology, synthetic biology, and personalized medicine. Cutting-edge computational tools, such as AI-driven drug design platforms, have accelerated the identification and optimization of antiviral compounds by using deep learning algorithms to predict the binding affinity of compounds to viral targets, thereby enhancing screening efficiency [12]. Network pharmacology, an emerging branch of systems biology, leverages multi-omics data to construct biological network models, uncovering how multiple components in natural products and traditional Chinese medicine (TCM) formulas synergistically interact with host cell signaling pathways to combat viral infections [13]. Moreover, advances in CRISPR-Cas9 gene editing technology have paved the way for novel antiviral strategies based on host gene regulation, such as modifying key host factors to bolster cellular defense mechanisms or disrupt the viral replication cycle [14]. Additionally, applications in synthetic biology have promoted the development of artificially synthesized virus-like particles (VLPs), which serve as vaccines or delivery vectors, carrying specific antigens to stimulate immune responses [15]. In the realm of personalized medicine, precision medicine utilizes big data analysis and machine learning techniques to comprehensively evaluate individual genotypes and clinical phenotypes, enabling the customization of treatment regimens tailored to patient-specific conditions, including real-time monitoring and adjustment of therapies in response to viral variants [16]. The convergence of these cutting-edge technologies not only deepens our understanding of viral pneumonia but also paves the way for safer and more effective therapeutic approaches.

This article aims to comprehensively summarize the current applications and limitations of antiviral drugs in the treatment of viral pneumonia while also evaluating the research progress of natural components, particularly plant extracts, in the management of viral pneumonia in recent years. We will analyze their potential mechanisms in antiviral therapy and discuss their prospects as adjunctive therapies. By reviewing this content, we hope to provide a scientific basis for future clinical treatment strategies, especially regarding the combined application of antiviral drugs and natural components.

## Antiviral drugs

For viral pneumonia, there are various antiviral drugs commonly used in clinical practice today. Treatment protocols are selected based on the type of virus and the specific circumstances of the patient, Table 1 is about Summary table of antiviral drugs.

**Table 1:** Summary table of antiviral drugs

Drug category	Drug name	Mechanism of action	Main indications	Common dosage	Advantages	Disadvantages	Special precautions	References
Broad-spectrum antiviral drugs	Ribavirin	Inhibits viral RNA polymerase, reduces intracellular GTP production, interferes with viral RNA and protein synthesis	Pneumonia caused by Respiratory Syncytial Virus (RSV)	Nebulization: 20 mg/mL, three times daily for 3–7 days; Oral: 600–800 mg, twice daily	Broad-spectrum antiviral activity against both RNA and DNA viruses Significant efficacy against RSV infections Can be administered via nebulization, suitable for children and critically ill patients	Dose-dependent anemia Teratogenic effects; contraindicated in pregnant women Long-term use may lead to viral resistance	Dose-dependent anemia, teratogenic effects, long-term use may lead to viral resistance	[20–24]
	Arbidol	Binds to viral hemagglutinin, prevents viral fusion with host cells, has immunostimulatory effects	Influenza and other respiratory viral infections	Oral: 200 mg, twice daily for 5–7 days	Broad-spectrum antiviral activity against multiple viruses Has immune-enhancing effects, boosting host resistance Convenient oral administration	Efficacy depends on early intervention May be affected by viral resistance or mutations Limited clinical evidence	Efficacy depends on early intervention, may be limited by viral resistance or mutation	[26–31]
Antiherpetic drugs	Acyclovir	Converts to the active form acyclovir triphosphate, competitively inhibits viral DNA polymerase, terminates viral DNA chain synthesis	Herpes Simplex Virus (HSV), Varicella-Zoster Virus (VZV) infections	Oral: 200 mg, every 4 h; Intravenous: adjusted based on patient condition	Significant efficacy against HSV and VZV infections Available as both oral and intravenous formulations Widely used in clinical practice with ample evidence	Risk of resistance Potential nephrotoxicity Limited effectiveness against influenza and coronaviruses	Resistance, nephrotoxicity, side effects, limited effectiveness against influenza and coronaviruses	[33–36]
	Ganciclovir	Converts to the active form ganciclovir triphosphate, competitively inhibits viral DNA polymerase, terminates viral DNA chain synthesis	Human Cytomegalovirus (CMV) infections, especially in immunocompromised patients	Intravenous: 5 mg/kg, once daily for 7–14 days	Significant efficacy against CMV infections Widely used in the treatment of immunocompromised patients	Can cause bone marrow suppression Nausea, liver function abnormalities Long-term use may lead to resistance	Bone marrow suppression, nausea, liver function abnormalities, may lead to resistance, limited effectiveness against HSV	[38–40]
Neuraminidase inhibitors (NAI)	Oseltamivir	Inhibits neuraminidase activity, prevents the release of viral particles from host cells	Influenza A and B	Treatment: 75 mg, twice daily for 5 days; Prophylaxis: 75 mg, once daily for 10 days	Convenient oral administration, suitable for self-management by patients with mild symptoms Widely used in clinical practice with ample evidence Low toxicity	Risk of resistance, particularly in H1N1 influenza A Must be used early (within 48 h of symptom onset)	Resistance, common side effects include nausea, vomiting, diarrhea, headache, optimal when administered within 48 h of symptom onset	[51–53]
	Zanamivir	Inhibits neuraminidase activity, prevents the release of viral particles from host cells	Mild to moderate influenza	Inhalation: 10 mg, twice daily for 5 days; Prophylaxis: 10 mg, once daily for 10 days	Effective against both influenza A and B Administered via inhalation, reducing systemic side effects Low risk of resistance	Inhalation administration limits its use in certain patients (e.g., those with breathing difficulties) Must be used early (within 48 h of symptom onset) May cause bronchospasm	Not suitable for patients with asthma or COPD, may cause throat irritation, coughing, bronchospasm	[55–58]
	Peramivir	Inhibits neuraminidase activity, prevents the release of viral particles from host cells	Severe influenza	Intravenous: 600 mg/kg, single dose	Intravenous administration, suitable for severe cases or patients unable to take oral medications Rapid onset of action, short course of treatment Low risk of resistance	Intravenous administration limits its use in outpatient settings Long-term safety and resistance issues not fully evaluated	Primarily used for hospitalized patients with severe influenza, less frequently used in mild cases	[60–62]
M2 ion channel blockers	Amantadine	Blocks the M2 ion channel, inhibits the entry of hydrogen ions into the viral particle, affects viral uncoating	Influenza A	**–**	Convenient oral administration Relatively inexpensive	High resistance rates, especially in H3N2 and H1N1 subtypes Potential central nervous system side effects (e.g., dizziness, insomnia) Not effective against influenza B	No longer recommended for clinical use due to high levels of resistance	[63–65]
	Rimantadine	Blocks the M2 ion channel, inhibits the entry of hydrogen ions into the viral particle, affects viral uncoating	Influenza A	**–**	Convenient oral administration Fewer central nervous system side effects compared to amantadine	Still has a high risk of resistance Not effective against influenza B Long-term safety needs further research	No longer recommended for clinical use due to high levels of resistance	
Interferons (IFN)	Interferon α/β/γ	Binds to specific receptors on cell surfaces, activates signaling pathways, induces the production of antiviral enzymes, enhances immune response	Various viral pneumonias, such as SARS-CoV-2 and influenza	Varies by type and condition, typically administered via nebulization or injection	Broad-spectrum antiviral activity Enhances host immune response Effective against multiple viruses	May cause severe systemic side effects (e.g., fever, fatigue, myalgia) Requires frequent injections Long-term safety concerns	Minimal severe side effects, but may cause flu-like symptoms such as fever and muscle pain	[65, 68]

### Broad-spectrum antiviral drugs

#### Ribavirin

Ribavirin is a guanosine analogue that exhibits broad-spectrum antiviral activity against both RNA and DNA viruses [17]. Ribavirin plays an important role in the treatment of viral pneumonia by inhibiting viral replication and dissemination. Upon entering infected cells, ribavirin is rapidly phosphorylated to form monophosphate, diphosphate, and triphosphate metabolites, which inhibit viral RNA polymerases, inosine monophosphate dehydrogenase, and mRNA guanylyltransferase, reducing the production of intracellular guanosine triphosphate (GTP). This interferes with the synthesis of viral RNA and proteins, ultimately inhibiting the assembly and release of viral particles [18, 19]. Ribavirin is less effective against influenza viruses and is primarily used in the antiviral treatment of viral pneumonias caused by respiratory syncytial virus (RSV) [20, 21]. For instance, nebulized formulations of ribavirin have been approved by the FDA for the treatment of RSV infections in infants, but are not indicated for adults [22]. The standard regimen involves administration at approximately 20 mg/mL, with inhalation three times daily over a course of 3–7 days. Oral administration of ribavirin (600–800 mg twice daily), with or without intravenous immunoglobulin (IVIG at 500 mg/kg every 48 h), represents a well-tolerated method for treating RSV infections in moderately to severely immunocompromised hosts [23]. Despite demonstrating significant efficacy in treating viral pneumonia, the toxic side effects of ribavirin cannot be ignored, such as dose-dependent anemia and teratogenic effects on fetuses. Long-term use can also lead to the emergence of viral resistance [24]. Therefore, treatment with ribavirin for viral pneumonia must be conducted under close monitoring to ensure effectiveness while minimizing adverse effects.

#### Hemagglutinin inhibitor: arbidol

Arbidol (ARB) is an indole-derived small molecule manufactured in Russia and licensed in Russia and China for the prevention and treatment of influenza and other respiratory viral infections [25]. ARB exhibits broad-spectrum antiviral activity against both RNA and DNA viruses and has a dual pharmacological effect, specifically acting on respiratory viruses while also having immunostimulatory properties, inducing serum interferon and activating phagocytes [26]. Hemagglutinin is a glycoprotein that facilitates the fusion of the virus with the host cell membrane, and arbidol blocks its binding to the host cell, preventing viral entry and thus inhibiting viral replication [27]. The drug demonstrates significant antiviral activity against various respiratory viral infections, such as influenza viruses and coronaviruses [28]. Arbidol works by binding to viral hemagglutinin, interfering with its fusion with host cell membrane receptors, thereby inhibiting viral entry into host cells and reducing the occurrence of pulmonary lesions [29]. The commonly used dosage is 200 mg per administration, taken twice daily for a continuous period of 5–7 days [30]. Although arbidol shows certain efficacy in treating viral pneumonias such as influenza, its primary target is RNA viruses, and its effectiveness depends on early intervention. The antiviral effect may be limited by viral resistance or mutation. Additionally, in cases of severe infection or rapid progression, the efficacy of arbidol may be inferior to combination therapy or other medications [31]. Therefore, its application requires a comprehensive evaluation based on the specific viral type and the patient’s condition.

### Antiherpetic drugs

#### Acyclovir

Acyclovir, also known as acyclic guanosine, is used to treat infections caused by Herpes Simplex Virus (HSV) and Varicella-Zoster Virus (VZV) [32]. Its mechanism of action involves converting to the active form acyclovir triphosphate, which competitively inhibits viral DNA polymerase, thereby blocking the synthesis of viral DNA and embedding itself into the viral DNA chain, causing chain termination and further inhibiting viral replication [33, 34]. Acyclovir can be administered orally or via intravenous injection. Common dosages involve taking 200 mg orally every four hours, with intravenous dosing adjusted according to the patient’s condition. While acyclovir is effective in treating herpesvirus infections, its use is limited by issues such as resistance, nephrotoxicity, and side effects, and it has weaker effects against other viruses like influenza and coronavirus [35, 36]. Therefore, its use requires a comprehensive assessment based on the specific condition of the patient.

#### Ganciclovir

Ganciclovir is primarily used to treat infections caused by Human Cytomegalovirus (CMV), especially in immunocompromised patients such as those with AIDS and organ transplant recipients [37]. Its mechanism of action involves converting to the active form ganciclovir triphosphate, which competitively inhibits viral DNA polymerase, blocking the synthesis of viral DNA, thereby inhibiting viral replication and causing chain termination by embedding itself into the viral DNA [38, 39]. Ganciclovir is primarily administered via intravenous injection, with a common dosage of 5 mg per kilogram of body weight once daily for 7–14 days. Although ganciclovir is effective in treating CMV infections, its use is accompanied by side effects such as bone marrow suppression, nausea, and liver function abnormalities, and it may lead to the development of resistance [40]. Ganciclovir is mainly effective against CMV and has weaker effects on other viruses such as Herpes Simplex Virus. Therefore, its application requires a comprehensive consideration of its side effects and the risk of resistance development.

### Neuraminidase inhibitors, NAIs

Neuraminidase inhibitors (NAIs) control influenza infections effectively by inhibiting the activity of neuraminidase on the surface of the influenza virus, thereby preventing the spread of the virus between host cells [41]. Neuraminidase is a key glycosidase on the surface of the influenza virus that is responsible for cleaving sialic acid residues from the surface of host cells, allowing the virus to be released from infected host cells and infect neighboring cells [42, 43]. NAI works by binding to neuraminidase, competitively inhibiting its activity and blocking the release of viral particles [44]. Specifically, after binding to neuraminidase, NAIs prevent the enzyme from interacting with sialic acid on the host cell surface, thus preventing newly produced viral particles from being released from the host cell. This mechanism not only reduces viral spread but also indirectly mitigates cellular damage and pathological changes caused by viral infection, promoting patient recovery [45]. Common NAIs include oseltamivir, zanamivir, and peramivir, which are clinically significant in the treatment of influenza virus infections [46]. Among these, oseltamivir is recommended by the World Health Organization (WHO) as the optimal clinical drug for combating influenza viruses, effective against both influenza A and B [47]. However, caution is needed regarding resistance and side effects, and early intervention following symptom onset is crucial for achieving optimal therapeutic outcomes.

#### Oseltamivir

As an important drug for treating and preventing influenza, oseltamivir is widely used [48–50]. For treatment, it is recommended to be taken within 48 h of the onset of flu symptoms. The adult dosage is 75 mg twice daily for five days. For prophylaxis during high flu seasons or following close contact with an infected individual, it is taken once daily at the same dose of 75 mg for ten days. One study showed that patients with influenza A and B who received oseltamivir had significantly shorter fever durations compared to those who did not receive antiflu medication (*P* values < 0.01) [51]. Patients with influenza B had longer times to fever resolution and fever duration after the first dose of oseltamivir compared to those with influenza A. However, the use of oseltamivir faces the issue of resistance, as some influenza virus strains may develop resistance to it [52]. Additionally, common side effects include nausea, vomiting, diarrhea, and headache, which may affect patient compliance. Furthermore, its efficacy is optimal when administered within 48 h of symptom onset, and delayed treatment may diminish its effectiveness [53].

#### Zanamivir

Zanamivir is suitable for the treatment of mild to moderate influenza in patients [54] and is administered via inhalation. The recommended dosage for adults is 10 mg per administration, given twice daily for five days. During influenza outbreaks, it can also be used for prophylaxis following exposure to an influenza patient, with a dosage of 10 mg once daily for ten days [55, 56]. However, because zanamivir is administered via inhalation, it may not be suitable for patients with respiratory conditions such as asthma or chronic obstructive pulmonary disease (COPD), thereby limiting its application [57]. Additionally, inhalation can cause throat irritation, coughing, and bronchospasm, which may lead some patients to discontinue use due to discomfort [58].

#### Peramivir

Peramivir is an intravenously administered antiflu drug primarily used for the treatment of severe influenza cases [59]. It is administered to hospitalized patients at a dose of 600 mg per kilogram of body weight in a single intravenous injection [60]. Although peramivir demonstrates good efficacy in severe influenza patients, its application is mainly limited to severe cases, with less frequent use in patients with mild influenza [61]. Additionally, common side effects include diarrhea, nausea, vomiting, and allergic reactions, which require monitoring during treatment [62].

### M2 ion channel blockers

Amantadine and rimantadine were once important drugs used to treat influenza viruses by blocking the function of the M2 ion channel, inhibiting the entry of hydrogen ions into the viral particle, thus affecting the uncoating process of the virus and ultimately inhibiting viral replication and spread [63, 64]. However, due to widespread high levels of resistance among influenza viruses, these drugs are no longer recommended for clinical use [65].

### Interferon, IFN

Interferons (IFNs) play an immunomodulatory role in the treatment of viral pneumonia. While IFNs do not directly kill viruses, they enhance the body’s antiviral capabilities by inducing the production of antiviral enzymes in host cells and boosting the immune response [66]. Specifically, interferons work by binding to specific receptors on the surface of cells, activating signaling pathways that promote the synthesis of antiviral proteins such as 2′,5′-oligoadenylate synthetase (OAS) and protein kinase R (PKR), which inhibit viral replication and spread [67]. Additionally, IFNs augment the activity of T cells and natural killer cells (NK cells), modulate B cell function, and increase antibody production, thereby enhancing the overall immune response. In the treatment of pneumonia caused by viruses such as SARS-CoV-2 and influenza, the application of interferons as immunomodulators can alleviate symptoms, shorten the duration of illness, and improve patient survival rates [68, 69]. Thus, interferons effectively bolster the body’s defenses against viral infections through multiple mechanisms, providing new strategies and directions for the treatment of viral pneumonia.

Current antiviral treatments for viral pneumonia have made significant progress, with various drug classes such as ganciclovir, NAIs, M2 ion channel blockers, and interferons demonstrating efficacy against specific types of viral infections. However, these drugs also face numerous challenges, including increased viral resistance due to long-term use, notable side effects like bone marrow suppression and liver dysfunction, and limited effectiveness against certain virus types. While existing drugs play a crucial role in controlling the condition, their limitations and potential risks restrict their broad application, especially when dealing with continuously mutating viruses.

To address these challenges, future research should focus on new drug development, combination therapies, personalized medicine, and preventive measures. By exploring novel antiviral drugs to overcome resistance issues, studying the synergistic effects of different drugs to enhance efficacy and reduce side effects, tailoring treatment plans based on individual patient characteristics, and strengthening preventive measures for viral pneumonia—such as developing more effective vaccines and increasing public health awareness—there is an expectation that safer, more effective, and sustainable solutions can be found. Through multidisciplinary collaboration and ongoing efforts, we aim to better tackle the global public health challenges posed by viral pneumonia.

## Natural components with antiviral activity

When discussing effective treatment strategies for viral pneumonia, besides traditional antiviral drugs, natural components are increasingly gaining attention as adjuvant therapies. These natural components not only possess potential antiviral activity but can,also enhance the body’s immune response, providing additional protection for patients [8, 70]. Table 2 is about natural compounds and their antiviral mechanisms. Here are several natural antiviral components that research has shown to be effective against viral pneumonia:

**Table 2:** Natural compounds and their antiviral mechanisms

Name	Chemical structure	Mechanism	Limitations	References
Astragaloside IV		Inhibits ROS production; inhibits NLRP3 inflammasome and Caspase-1 activation; reduces IL-1β and IL-18 secretion; inhibits TGF-β1/Smad pathway	The specific mechanisms, dose dependency, safety, and synergistic effects with conventional antiviral therapies require further basic research and clinical validation	[73–75]
Houttuynia Cordata Flavonoids	**–**	Inhibits influenza virus and Toll-like receptor signaling; regulates gut microbiota, reduces pathogenic Enterobacteriaceae and pro-inflammatory cytokines	Shows better efficacy when used in combination with polysaccharides; its effectiveness as a standalone treatment may be limited and requires more research to determine optimal usage	[78–82]
Houttuynia Cordata Polysaccharides	**–**	Regulates gut microbiota, reduces pathogenic Enterobacteriaceae and pro-inflammatory cytokines	Its effectiveness is less pronounced when used alone compared to when combined with flavonoids, and its exact mechanisms are not fully understood	
Theaflavin-3′-Gallate		Inhibits influenza virus replication; inhibits TLR4/MAPK/p38 signaling pathway	Limited direct evidence for use in viral pneumonia; more experimental and clinical studies are needed to validate its efficacy	[85, 86]
Berberine		Inhibits influenza virus replication; inhibits TLR7 signaling pathway; inhibits NLRP3 inflammasome activation	May cause gastrointestinal side effects; long-term safety and efficacy need further evaluation	[89, 90]
Paeoniflorin		Alleviates acute lung injury; inhibits TGF-β1, Smad2, NF-κB, and p38MAPK expression	Research on its application in viral pneumonia is relatively limited; more targeted studies are needed	[92–94]
Patchouli Alcohol		Prevents viral membrane fusion with intracellular membranes; inhibits influenza virus replication; enhances host immune response	Lacks large-scale clinical trial data on its specific application in viral pneumonia	[96–99]
Emodin		Inhibits multiple viral infections; inhibits influenza virus replication; activates Nrf2 pathway, reduces ROS levels	Long-term toxicity and safety issues are not fully established; caution is advised in its use	[103, 104]
Resveratrol		Inhibits RSV-induced persistent airway inflammation; reduces inflammatory cells and NGF levels	High doses may cause adverse reactions like gastrointestinal discomfort; low bioavailability affects efficacy	[106]
Geniposide		Inhibits lung inflammation, activates Nrf2, inhibits NF-κB; inhibits nuclear export of influenza virus gene mRNAs, reduces M1 protein expression	Most research focuses on liver diseases; specific efficacy for viral pneumonia needs more study	[108–112]
Glycyrrhizic Acid		Increases nitric oxide production; alters viral lipid bilayer, binds to ACE2 receptor; inhibits HMGB1/TLR4 signaling pathway	May cause water and sodium retention and increased blood pressure, making it unsuitable for all patients	[114–119]
Baicalein		Inhibits SARS-CoV-2 replication; inhibits influenza virus replication, inhibits neuraminidase activity	Low bioavailability and poor oral absorption limit its clinical application	[123, 124]

### Astragalus IV

Astragalus membranaceus is one of the most widely used traditional Chinese herbs. It serves as an immunostimulant, tonic, antioxidant, hepatoprotectant, diuretic, antidiabetic, anticancer, and expectorant [71, 72]. Astragaloside IV (AS-IV), the most active monomer in Astragalus, exhibits extensive antiviral, anti-inflammatory, and antifibrotic pharmacological effects, showing protective effects in acute lung injury [73]. Studies have shown that AS-IV inhibits the production of reactive oxygen species (ROS) in a dose-dependent manner in A549 cells infected with influenza virus, thereby inhibiting the activation of the NOD-like receptor pyrin domain-containing 3 (NLRP3) inflammasome and Caspase-1, reducing the secretion of interleukin (IL)-1β and IL-18. In BALB/c mice infected with Poly(I:C), oral administration of AS-IV significantly alleviates Poly(I:C)-induced acute pneumonia and pulmonary pathology [74]. Additionally, astragaloside can reduce lung inflammation in rats by inhibiting the TGF-β1/Smad pathway [75]. This indicates that astragaloside has potential in the treatment of viral pneumonia.These studies indicate that Astragalus membranaceus and its active component, AS-IV, may be a significant source for developing new antiviral drugs or as an adjuvant therapy for viral respiratory diseases. They not only enhance immune system function but also provide direct protective effects during viral infections, mitigating inflammatory responses and tissue damage.

### Houttuynia cordata flavonoids and polysaccharides

Houttuynia cordata is a classic Traditional Chinese Medicine (TCM) used clinically for the treatment of pneumonia [76, 77]. Total flavonoids (HCF) and polysaccharides (HCP) are key medicinal components of Houttuynia cordata in the treatment of viral pneumonia [78]. Research has shown that flavonoids in Houttuynia cordata can alleviate acute lung injury induced by H1N1 in mice by inhibiting influenza virus and Toll-like receptor signaling [79]. For lethal H1N1 infections in mice, the combination of HCF and HCP significantly increased survival rates and extended lifespan compared to monotherapy. The combined use of HCF and HCP can markedly improve symptoms of viral pneumonia, manifested by reduced lung indices, more intact lung tissue morphology, decreased inflammatory cells and mediators. Moreover, the combination of HCF and HCP can regulate gut microbiota, significantly reducing the proportion of pathogenic Enterobacteriaceae and pro-inflammatory cytokine secretion, demonstrating synergistic effects in reducing lung and gut damage [80, 81]. A 70% ethanol extract from the aerial parts of Houttuynia cordata inhibited the production of inflammatory biomarkers IL-6 and NO in lung epithelial cells (A549) and alveolar macrophages (MH-S). Oral administration of the same plant material (100 and 400 mg/kg) significantly inhibited the pulmonary inflammatory response in a lipopolysaccharide (LPS)-induced acute lung injury model in mice. Major flavonoid compounds were successfully isolated from the extract, which also alleviated LPS-induced pulmonary inflammation in mice when administered orally [82]. Additionally, studies on the effects of flavonoids from Houttuynia cordata revealed that hyperoside, quercitrin, and quercetin were more effective against H1N1 infection than HCF [83]. This suggests that the combination of flavonoids and polysaccharides from Houttuynia cordata holds advantages in the treatment of viral pneumonia. These research findings indicate that Houttuynia cordata and its active components, particularly flavonoids and polysaccharides, show promising potential in the treatment of viral pneumonia. They not only enhance immune system function but also directly combat viral infections, reduce inflammatory responses, and further protect lung and gut health by modulating the gut microbiota. However, although the preliminary results are very encouraging, more clinical trials are needed to verify the safety and efficacy of Houttuynia cordata and its extracts before they can be applied in clinical treatments.

### Theaflavin-3′-gallate

Theaflavin-3′-gallate (T3G) is a monomer of theaflavins found in black tea and is considered an important bioactive component beneficial to health [84]. Theaflavin-3′-gallate (T3G) and theaflavin (TF1) can effectively inhibit the replication of influenza viruses such as H1N1-UI182, H1N1-PR8, H3N2, and H5N1, with T3G demonstrating the most significant antiviral activity in vivo. Intraperitoneal injection of 40 mg/kg/day T3G effectively alleviated viral pneumonia, maintained body weight, and increased the survival rate of mice infected with a lethal dose of H1N1-UI182 to 55.56%. Peripheral blood hematological analysis further showed that T3G increased lymphocyte counts and decreased neutrophil, monocyte, and platelet counts in infected mice. RT-qPCR results indicated that T3G reduced the mRNA expression levels of inflammatory cytokines (IL-6, TNF-α, and IL-1β), chemokines (CXCL-2 and CCL-3), and interferons (IFN-α and IFN-γ) post-influenza virus infection. Additionally, T3G significantly downregulated the expression levels of TLR4, p-p38, p-ERK, and cytokines IL-6, TNF-α, IL-1β, and IL-10 [85]. Studies indicate that T3G binds tightly to the SARS-CoV-2 spike protein RBD with a very low KD value of 1.3 nM, suggesting it could disrupt ACE2 binding, potentially preventing viral entry. T3G also blocks the main protease (Mpro) of SARS-CoV-2 with an IC50 of 18.48 μM and has been shown to reduce viral load by 75% under laboratory conditions [86]. These findings suggest that T3G not only significantly inhibits viral replication and proliferation in vitro but also mitigates pneumonia damage in vivo. Its antiviral effects may be attributed to the downregulation of influenza virus-induced inflammatory cytokines via modulation of the TLR4/MAPK/p38 signaling pathway.These research findings indicate that T3G has potential applications in combating a variety of viruses, including influenza viruses and SARS-CoV-2. It not only exhibits strong antiviral activity but also reduces inflammatory damage by modulating the immune response.

### Berberine

Berberine is a natural isoquinoline alkaloid isolated from plants of the Berberis genus, known for its diverse biological properties including anti-inflammatory, antibacterial, antifungal, and anthelmintic effects [87, 88]. Studies have shown that berberine strongly inhibits the replication of Influenza A virus A/FM1/1/47 (H1N1) in A549 cells and in mouse lungs. Additionally, berberine alleviated lung inflammation in mice compared to those treated with a vehicle, reducing necrosis, inflammatory cell infiltration, and pulmonary edema caused by viral infection. Berberine suppressed the upregulation of the TLR7 signaling pathway (such as TLR7, MyD88, and NF-κB (p65)) induced by viral infection at both the mRNA and protein levels. Furthermore, berberine significantly inhibited the increase in the Th1/Th2 and Th17/Treg ratios and the production of inflammatory cytokines induced by viral infection [89]. These findings suggest that berberine may improve lung inflammation in mice with influenza viral pneumonia by inhibiting NLRP3 inflammasome activation and pyroptosis mediated by GSDMD. Another study indicates that berberine primarily exerts therapeutic effects on lung fibrosis associated with COVID-19 pneumonia by regulating cell proliferation, metabolism, and survival via tumor necrosis factor-alpha (TNF-α), interleukin-6 (IL-6), signal transducer and activator of transcription 3 (STAT3), and chemokine (C–C motif) ligand 2 (CCL2) [90]. In mouse experiments, berberine reduced the mortality rate from 90 to 55% and decreased the viral titer in the lungs two days post-infection (*P* < 0.05). Berberine also improved lung histopathology, with significantly lower lung histological scores on the 2nd, 4th, and 6th days of treatment compared to the untreated group (*P* < 0.05). Additionally, it effectively inhibited the generation of NO and iNOS (*P* < 0.01) and reduced the expression of TNF-α on day 4 (*P* < 0.01) and day 6 (*P* < 0.05), as well as suppressing MCP-1 expression on day 6 (*P* < 0.01), demonstrating its anti-inflammatory effects [91].These results indicate that Berberine combats viral infections through multiple mechanisms. It not only demonstrates strong antiviral activity but also reduces inflammatory damage by modulating the immune response.

### Paeoniflorin

Paeoniflorin is an herbal component derived from the root of Paeonia lactiflora. It is used in traditional Chinese medicine for its antispasmodic and analgesic properties and is known for its anticoagulant, neuromuscular blocking, cognitive enhancement, immunomodulatory, and antihyperglycemic effects [92]. Paeoniflorin (at doses of 50 and 100 mg/kg) can alleviate acute lung injury induced by Influenza A Virus (IAV). It reduces pulmonary edema, improves lung tissue pathology, and decreases the accumulation of inflammatory cells in the lungs. Results show that paeoniflorin (50 and 100 mg/kg) can ameliorate acute lung injury induced by IAV, increasing the survival rate of infected mice (by 40% and 50%, respectively), lowering the viral titer in lung tissues, improving histological changes, and reducing lung inflammation. Paeoniflorin also decreases the levels of pulmonary fibrosis markers (type IV collagen, α-smooth muscle actin, hyaluronic acid, laminin, and procollagen III) and downregulates the expression levels of type I collagen (Col I) and type III collagen (Col III) in lung tissues, thus improving pulmonary fibrosis. Additionally, paeoniflorin inhibits the expression of αvβ3, TGF-β1, Smad2, NF-κB, and p38MAPK in lung tissues [93].

The combination of carboxymethyl chitosan and dextran sulfate (CS-DS) exhibited synergistic effects in lipopolysaccharide (LPS)-induced acute lung injury (ALI). Two compounds identified through network pharmacology screening, paeoniflorin and luteolin, significantly reduced the expression levels of reactive oxygen species (ROS), nitric oxide (NO), tumor necrosis factor-alpha (TNF-α), interleukin-6 (IL-6), and interleukin-1β (IL-1β) in LPS-stimulated RAW264.7 cells. In the LPS-induced ALI model, the combination of paeoniflorin and luteolin similarly reduced the expression of inflammatory factors and oxidative stress levels. Furthermore, the expression of proteins involved in the LPS-activated NF-κB and MAPK signaling pathways was effectively inhibited by this combination therapy [94].Paeoniflorin demonstrates significant efficacy in combating viral infections and acute lung injury through multiple mechanisms. Not only does it directly inhibit viral replication, but it also protects lung health by modulating immune responses, reducing inflammation, and improving pulmonary fibrosis. Particularly, the combined use of Paeoniflorin and Luteolin in LPS-induced acute lung injury models exhibits stronger anti-inflammatory and antioxidant effects, suggesting that this combination therapy may become an effective strategy for treating acute lung injury in the future.

### Patchouli alcohol

Patchoulol is a tricyclic sesquiterpene extracted from Agastache rugosa and has been traditionally used in Chinese medicine to treat inflammatory conditions [95]. Patchoulol exhibits various pharmacological activities, including antiemetic, anti-inflammatory, antibacterial, and antiviral properties [96]. It has been confirmed that patchoulol can interact specifically with HA2, preventing the fusion of the viral membrane with intracellular membranes, thereby inhibiting the early life cycle of influenza A viruses [97]. When evaluated for its anti-influenza virus activity against A/PR/8/34 using a plaque formation assay, patchoulol reduced plaque numbers by 75% at 2 μg/mL and by 89% at 10 μg/mL. Patchoulol demonstrates dose-dependent antiviral activity against influenza A viruses, with an estimated IC50 value of 2.635 μM [98]. Research indicates that the oral administration of patchoulol appears to enhance protection against influenza virus infection in mice by boosting the host’s immune response and dampening systemic and pulmonary inflammatory responses. Patchoulol primarily inhibits influenza A (H2N2) virus by interfering with the function of viral neuraminidase. It was found that patchoulol can inhibit the replication of different influenza A viruses in vitro, with pandemic H1N1 virus (Vir09) being the most sensitive to patchoulol treatment (IC50 < 6.5 μg/mL). Compared to the positive control drug oseltamivir, intranasal administration of patchoulol significantly improved the survival rate of mice and alleviated pneumonia symptoms in influenza A virus (IAV)-infected mice. Patchoulol exhibits anti-IAV activity both in vivo and in vitro and may block IAV infection by targeting viral particles and cellular PI3K/Akt and ERK/MAPK signaling pathways [99]. Therefore, patchoulol is worthy of further investigation as a novel anti-IAV drug candidate in future research.Patchoulol demonstrates considerable potential in combating influenza viruses, particularly in inhibiting viral replication, enhancing immune responses, and reducing inflammation. Therefore, Patchoulol warrants further in-depth research as a novel candidate for anti-IAV drugs to evaluate its feasibility and safety for clinical applications.

### Emodin

Emodin (1,3,8-trihydroxy-6-methylanthraquinone) is a natural anthraquinone compound [100], derived from various traditional Chinese medicinal plants such as Polygonum multiflorum, Rheum palmatum, and Polygonum cuspidatum, known for its antioxidant, anti-inflammatory, immunosuppressive, antiviral, and antitumor activities [101, 102]. Emodin also inhibits infections by Coxsackie virus (CV), human respiratory syncytial virus (RSV), Epstein-Barr virus (EBV), and hepatitis B virus (HBV) [103]. Studies have shown that emodin can significantly inhibit the replication of Influenza A Virus (IAV, ST169, H1N1), decrease IAV-induced expression of TLR2/3/4/7, MyD88, and TRAF6, and reduce IAV-induced phosphorylation of p38/JNK MAPK and nuclear translocation of NF-κB p65. Emodin can also activate the Nrf2 pathway, lower ROS levels, increase GSH levels and the GSH/GSSG ratio, and upregulate the activity of SOD, GR, CAT, and GSH-Px. Knockdown of Nrf2 by siRNA significantly blocks the inhibitory effect of emodin on IAV-induced activation of the TLR4, p38/JNK, and NF-κB pathways, as well as IAV-induced production of IL-1β, IL-6, and IAV M2 protein expression. Emodin also significantly increased the survival rate of mice, alleviated pulmonary edema and lung viral titers, and improved lung histopathological changes [104]. Additionally, emodin can significantly inhibit IAV replication and IAV-mediated inflammation, with its mechanism possibly related to the activation of the Nrf2 signaling pathway and inhibition of IAV-induced oxidative stress, TLR4, p38/JNK MAPK, and NF-κB pathway activation [104]. Emodin demonstrates extensive potential in combating influenza virus infections, particularly in inhibiting viral replication, modulating immune responses, and reducing inflammation. It exerts its antiviral and antioxidant effects by activating the Nrf2 pathway and inhibiting multiple inflammation-related signaling pathways, such as TLR4, p38/JNK, and NF-κB. Therefore, Emodin, as a novel candidate for anti-IAV drugs, warrants further in-depth research to evaluate its feasibility and safety for clinical applications.

### Resveratrol

Resveratrol, an antioxidant phytoalexin found in red grapes, has chemopreventive and therapeutic effects on various diseases [105]. Resveratrol significantly affects the persistent airway inflammation and airway hyperresponsiveness (AHR) induced by RSV infection. Mice infected with RSV are euthanized at consecutive time points post-infection to collect samples and measure the number of inflammatory cells and levels of interferon-gamma (IFN-γ), nerve growth factor (NGF), and brain-derived neurotrophic factor (BDNF). The administration of resveratrol is followed by assessments of airway inflammation, AHR, as well as NGF and BDNF levels. Additionally, anti-NGF antibodies (Ab-NGF) are used to investigate the role of NGF in the persistent airway inflammation and AHR induced by RSV. The study found that RSV RNA could still be detected in the lungs of RSV-infected mice on day 60, accompanied by persistent airway inflammation and AHR lasting 60 days. Levels of IFN-γ in the bronchoalveolar lavage fluid (BALF) increased on day 7 post-RSV infection but returned to normal levels by day 14 post-infection, while levels of NGF and BDNF gradually increased from day 14 to day 60. Furthermore, treatment with resveratrol led to a reduction in the total cell count in the BALF; the number of inflammatory cells infiltrating the lungs was also lower. Resveratrol attenuated the airway response to acetyl-methacholine and significantly lowered NGF levels in the BALF without affecting BDNF levels. Additionally, administration of Ab-NGF after RSV infection diminished the associated persistent airway inflammation and AHR. Resveratrol inhibits persistent airway inflammation and AHR, potentially in part by reducing NGF levels post-RSV infection [106]. Resveratrol demonstrates significant potential in combating the persistent airway inflammation and AHR caused by RSV infection, particularly by reducing NGF levels to exert its anti-inflammatory and immunomodulatory effects.

### Geniposide

Geniposide is an effective iridoid glycoside extracted from Forsythia suspensa predominantly found in the roots, stems, leaves, and fruits of the plant [107]. In terms of medicinal applications, geniposide exhibits pharmacological effects such as anti-inflammatory, weight loss, and anti-tumor activities in vitro and in animal models [108]. Previous studies have found that geniposide inhibits cigarette smoke-induced lung inflammation by activating Nrf2 and inhibiting NF-κB [109]. Similarly, geniposide can alleviate LPS-induced lung inflammation in acute lung injury mice by inhibiting the activation of MAPK and NF-κB [110]. Geniposide A can inhibit M1 expression but does not affect NP expression. This compound blocks the nuclear export of some influenza virus gene mRNAs at the post-transcriptional level, leading to differential production of viral proteins. Another possible mechanism for the reduction in M1 levels is enhanced protein degradation. Cyclophilin A, a member of the cyclophilin family and a peptidyl-prolyl isomerase, has been shown to accelerate the degradation of M1 protein via a ubiquitin/proteasome-dependent pathway, thereby inhibiting influenza virus replication [111]. In vitro studies have shown that, compared to the viral group, the expression levels of NF-κB p65, p-NF-κB p65 proteins were significantly reduced, and the expression levels of p-IκBα were significantly decreased, while the expression levels of IκBα were significantly increased in Huh-7 cells treated with KD-1 (250, 125, 62.5 μg/ml) [112]. This suggests that geniposide A has potential therapeutic effects in inhibiting influenza virus replication. Geniposide demonstrates extensive potential in anti-inflammatory and anti-influenza virus applications. It reduces inflammatory responses by activating the Nrf2 pathway and inhibiting the NF-κB pathway, and it suppresses influenza virus replication by blocking the nuclear export of viral mRNAs and promoting the degradation of the M1 protein.

#### Glycyrrhizic acid

Glycyrrhizin, also known as glycyrrhizic acid or licorice sweetening glycoside, is a triterpenoid saponin mainly isolated from the roots of the Glycyrrhiza glabra plant [113]. There are several proposed antiviral mechanisms for glycyrrhizin: increasing nitric oxide production in macrophages, affecting transcription factors and cellular signaling pathways, directly altering the viral lipid bilayer, and binding to the ACE2 receptor [114]. Glycyrrhizin influences cellular signaling pathways such as protein kinase C and casein kinase II, as well as transcription factors like activator protein 1 and nuclear factor kappa B. Additionally, glycyrrhizin and its metabolite 18β-glycyrrhetinic acid upregulate the expression of inducible nitric oxide synthase and nitric oxide production in macrophages. Preliminary results indicate that glycyrrhizin induces nitric oxide synthase in Vero cells, and when a nitric oxide donor (BETA NONOate) is added to the culture medium, viral replication is inhibited [115]. This suggests that glycyrrhizin has potential applications in antiviral treatments. Glycyrrhizin is considered a potential therapeutic agent for the novel coronavirus (COVID-19) [116]. In the context of SARS, oral doses of up to 300 mg and intravenous doses of approximately 240 mg have been recommended [117, 118]. Furthermore, research has found that glycyrrhizin can alleviate acute lung injury by inhibiting the HMGB1/TLR4 signaling pathway [119]. Glycyrrhizin demonstrates extensive potential in antiviral and anti-inflammatory applications, particularly in inhibiting viral replication, modulating immune responses, and reducing lung inflammation. Therefore, glycyrrhizin, as a novel antiviral drug candidate, warrants further research to evaluate its feasibility and safety for clinical applications.

#### Baicalein

Scutellaria baicalensis is a traditional Chinese medicine used for treating common colds, fevers, and influenza virus infections [120, 121]. Flavonoids baicalein and baicalin have demonstrated potent antiviral activity against SARS-CoV-2 in vitro [122]. Research has shown that baicalin can alleviate LPS-induced pulmonary inflammation via the NF-κB and MAPK pathways [123]. In vitro experiments have indicated that baicalin has a half-maximal effective concentration (EC50) of 43.3 μg/ml against influenza virus A/FM1/1/47 (H1N1) and a half-maximal inhibitory concentration (IC50) of 104.9 μg/ml against influenza virus A/Beijing/32/92 (H3N2). When added to MDCK cell cultures post-inoculation with influenza virus, the antiviral activity of baicalin was significantly increased in a dose-dependent manner, indicating that baicalin affects viral budding. Baicalin also exhibits a marked inhibitory effect on neuraminidase, with an IC50 of 52.3 μg/ml against influenza virus A/FM1/1/47 (H1N1) and an IC50 of 85.8 μg/ml against influenza virus A/Beijing/32/92 (H3N2). In vivo studies have shown that intravenous administration of baicalin can effectively reduce the mortality rate of mice infected with influenza A viruses, extend the mean death day (MDD), and improve lung parameters. These results suggest that baicalin acts as a neuraminidase inhibitor with significant inhibitory activity, effectively combating different strains of influenza A viruses in both cell culture and mouse models, indicating potential utility in the management of influenza virus infections [124]. Scutellaria baicalensis and its active component baicalin demonstrate extensive potential in antiviral and anti-inflammatory applications, particularly in inhibiting viral replication, modulating immune responses, and reducing lung inflammation. Therefore, baicalin, as a novel candidate for anti-influenza virus drugs, warrants further research to evaluate its feasibility and safety for clinical applications.

Natural components are gradually gaining attention in the treatment of viral pneumonia due to their diverse biological activities. These components not only potentially have direct antiviral effects but can also enhance the body’s immune response, reduce inflammation, and protect tissues from damage, providing additional protection for patients. Although natural components cannot completely replace traditional antiviral drugs, their role in comprehensive treatment regimens is becoming increasingly important. Traditional antiviral drugs face challenges such as increased drug resistance, more toxic side effects, and limited efficacy against certain types of viruses. The multi-target intervention approach provided by natural components can serve as a complementary therapy, reducing the limitations of using single drugs. Moreover, natural components typically have lower toxicity and fewer adverse reactions, making them safer for long-term use.

To better utilize natural components in combating viral pneumonia, future research should emphasize a deeper understanding of how these components affect viral replication, host immune responses, and inflammatory regulation. Large-scale clinical trials should be conducted to evaluate their safety and effectiveness, ensuring reliability and consistency in human applications. It is also crucial to develop combined treatment strategies that integrate natural components with existing antiviral medications to maximize synergistic effects and improve therapeutic outcomes. Additionally, applying the concept of precision medicine to explore personalized treatment plans for different types of viral pneumonia, tailoring the most suitable methods based on individual patient characteristics, can further enhance treatment efficacy. Through interdisciplinary collaboration and continuous effort, we aim to find safer, more effective, and sustainable solutions to address the global public health challenges posed by viral pneumonia.

## Limitations

Although this review provides a detailed exploration of the applications and potential mechanisms of antiviral drugs and natural compounds in the treatment of viral pneumonia, it also has several limitations. Specifically, while some potential mechanisms of natural compounds are mentioned—such as astragaloside IV reducing inflammation by inhibiting ROS production, NLRP3 inflammasome, and Caspase-1 activation, and flavonoids from Houttuynia cordata regulating gut microbiota to decrease pathogenic Enterobacteriaceae and pro-inflammatory cytokines—the specific mechanisms, dose-dependency, safety, and synergistic effects with traditional antiviral therapies of these compounds still require further basic research and clinical validation. This indicates that more scientific evidence is needed to establish the actual efficacy and safe use of these compounds.

Moreover, while natural compounds show potential as adjunctive treatments, they cannot fully replace conventional antiviral drugs, and integrating these compounds into existing treatment protocols remains challenging, including determining optimal combination therapies and indications. Additionally, the issues of drug resistance and increased toxicity associated with current antiviral drugs limit their broad application, and addressing these problems requires the development of new drugs or improvements to existing ones, which falls outside the scope of this review. Finally, regarding the application of natural compounds, the review emphasizes the need for larger-scale clinical trials to evaluate their safety and effectiveness; currently, evaluations are primarily based on laboratory studies or small-scale preliminary clinical observations, lacking sufficient clinical evidence to support their widespread clinical application. Therefore, future research should focus on deepening the understanding of the mechanisms of natural compounds, conducting large-scale clinical trials, exploring the possibilities of personalized medicine, and addressing the issues of drug resistance and toxicity associated with current antiviral drugs.

## Conclusion

This review comprehensively examines the current status of antiviral therapies for viral pneumonia, focusing on the advantages and limitations of existing antiviral drugs, while summarizing the potential roles of various natural compounds in the treatment of viral pneumonia. Existing antiviral drugs, such as ribavirin and arbidol, although effective against certain viral infections, face challenges due to rising resistance, increased toxic side effects, and limited efficacy, which restrict their widespread application. Natural compounds, owing to their multiple biological activities including antiviral, anti-inflammatory, immunomodulatory, and tissue-protective effects, have shown promise particularly in alleviating viral lung injury. However, the mechanisms of action, dose dependency, safety, and synergistic effects of these natural compounds alongside conventional antiviral therapies still require extensive fundamental research and clinical validation.

The treatment of viral pneumonia continues to face numerous challenges, especially with the increasing emergence of viral mutations and resistance, making it difficult for current therapeutic approaches to provide comprehensive solutions. Future research directions should emphasize: (1) a deeper understanding of the mechanisms by which natural compounds affect viral replication, host immune responses, and inflammation regulation; (2) larger-scale clinical trials to evaluate the safety and efficacy of these natural compounds; (3) the development of combination therapy strategies that integrate natural compounds with existing antiviral drugs to maximize synergistic effects; and (4) the application of precision medicine concepts to explore personalized treatment regimens tailored to different types of viral pneumonia.

Through such research efforts, natural compounds have the potential to complement traditional antiviral drugs, offering safer and more effective treatment options for viral pneumonia, reducing the impact of resistance and drug-related toxic side effects, thereby improving long-term patient outcomes.

## Data Availability

No datasets were generated or analysed during the current study.

## Abbreviations

HSV: Herpes simplex virus
NAI: Neuraminidase inhibitors
RSV: Respiratory syncytial virus
IFN: Interferons
CMV: Human cytomegalovirus
SARS-CoV-2: Severe acute respiratory syndrome coronavirus 2
M2: M2 ion channel
COPD: Chronic obstructive pulmonary disease
ACE2: Angiotensin-converting enzyme 2
PRRs: Pattern recognition receptors
TLRs: Toll-like receptors
RIG-I: Retinoic acid-inducible gene I
MDA5: Melanoma differentiation-associated protein 5
NLRs: NOD-like receptors
NLRP3: NOD-like receptor protein 3
caspase-1: Cysteine-aspartic protease 1
